# The Transcription Factor *HOXA5*: Novel Insights into Metabolic Diseases and Adipose Tissue Dysfunction

**DOI:** 10.3390/cells12162090

**Published:** 2023-08-18

**Authors:** Luca Parrillo, Rosa Spinelli, Michele Longo, Federica Zatterale, Gianluca Santamaria, Alessia Leone, Michele Campitelli, Gregory Alexander Raciti, Francesco Beguinot

**Affiliations:** 1 URT Genomics of Diabetes, Institute of Experimental Endocrinology and Oncology, National Research Council, Department of Translational Medical Sciences, Federico II University of Naples, 80131 Naples, Italy; spinelli.rossella@gmail.com (R.S.); mi_longo@libero.it (M.L.); federicazatterale@libero.it (F.Z.); aleleone86@libero.it (A.L.); m.campitelli@ieos.cnr.it (M.C.); gregoryraciti@gmail.com (G.A.R.); 2 Lundberg Laboratory for Diabetes Research, Department of Molecular and Clinical Medicine, Sahlgrenska Academy, University of Gothenburg, 41345 Gothenburg, Sweden; 3 Department of Experimental and Clinical Medicine, University Magna Græcia of Catanzaro, 88100 Catanzaro, Italy; gsantamaria@unicz.it

**Keywords:** *HOX* genes, adipose tissue dysfunction, metabolic diseases, obesity and type 2 diabetes, hypertrophic obesity, adipogenesis, fat mass distribution, inflammation, epigenetics, DNA methylation, epigenetic biomarker, targeted therapy

## Abstract

The transcription factor *HOXA5*, from the *HOX* gene family, has long been studied due to its critical role in physiological activities in normal cells, such as organ development and body patterning, and pathological activities in cancer cells. Nonetheless, recent evidence supports the hypothesis of a role for *HOXA5* in metabolic diseases, particularly in obesity and type 2 diabetes (T2D). In line with the current opinion that adipocyte and adipose tissue (AT) dysfunction belong to the group of primary defects in obesity, linking this condition to an increased risk of insulin resistance (IR) and T2D, the *HOXA5* gene has been shown to regulate adipocyte function and AT remodeling both in humans and mice. Epigenetics adds complexity to *HOXA5* gene regulation in metabolic diseases. Indeed, epigenetic mechanisms, specifically DNA methylation, influence the dynamic *HOXA5* expression profile. In human AT, the DNA methylation profile at the *HOXA5* gene is associated with hypertrophic obesity and an increased risk of developing T2D. Thus, an inappropriate *HOXA5* gene expression may be a mechanism causing or maintaining an impaired AT function in obesity and potentially linking obesity to its associated disorders. In this review, we integrate the current evidence about the involvement of *HOXA5* in regulating AT function, as well as its association with the pathogenesis of obesity and T2D. We also summarize the current knowledge on the role of DNA methylation in controlling *HOXA5* expression. Moreover, considering the susceptibility of epigenetic changes to reversal through targeted interventions, we discuss the potential therapeutic value of targeting *HOXA5* DNA methylation changes in the treatment of metabolic diseases.

## 1. Introduction

HOX genes are the largest and most extensively studied members of the major Homeobox transcription factor (TF) superfamily, which plays a significant role in embryonic development, hematopoiesis, and tumorigenesis [1]. The HOX gene family consists of 39 genes clustered in four chromosomal regions (HOXA, HOXB, HOXC, and HOXD). HOX genes encode TFs that bind via their homeodomain DNA motifs in HOX-responsive elements to regulate tissue-specific gene expression and thus direct the morphogenetic events leading to complex body forms [2]. Consequently, any mutations and unregulated expression of the HOX genes can lead to anomalies at various stages of development, causing aberrant phenotypes in humans [3,4].

The transcription factor *HOXA5* was initially identified because of its essential role in axial skeleton formation during development [5,6]. Subsequently, the analysis of *HOXA5* mutant mice has revealed a plethora of phenotypes, including organ defects and postnatal abnormalities, which are indicative of the broad range of actions of this gene throughout life. Indeed, an aberrant *HOXA5* expression contributes to anomalies and dysfunction in various organs, such as the thyroid gland, mammary glands, and ovaries, as well as affecting proliferation, differentiation, invasion, apoptosis, and other biological processes in multiple cancer types [5,6], where *HOXA5* can function as both an oncogene and tumor suppressor [7].

Besides this well-described role in normal development and carcinogenesis, increasing evidence further indicates that *HOXA5* is highly expressed in the adipose tissue (AT) and plays an active role in regulating adipocyte biology and body fat distribution [8]. Changes in *HOXA5* expression correlate with variations in adiposity levels and the pattern of body fat distribution [9]. Furthermore, extreme weight loss in obese humans is associated with the upregulation of the *HOXA5* gene in the subcutaneous AT (SAT), which implicates this transcriptional regulator in the expansion of AT [10,11]. The ability to recruit new adipocytes through adipogenesis is critical for a healthy AT expansion and systemic metabolic health in the setting of caloric excess [12]. We demonstrated that *HOXA5* is essential for proper adipogenesis in humans and mice [11,13], which would make it an appealing molecular target for the treatment of metabolic pathologies related to AT dysfunction since modulating adipogenesis is now emerging as a viable therapeutic strategy to promote healthy AT remodeling and an improved metabolic state during overnutrition [14].

Epigenetics adds a further layer of complexity to *HOXA5* gene regulation in relation to metabolic diseases [15,16]. DNA methylation is the most common strategy for the control of the dynamic *HOXA5* expression profile [5,6]. Indeed, an increased methylation in the *Hoxa5* promoter region contributes to its downregulation in the AT of mice exposed to a high-fat diet [11]. In addition, increase in methylation at the *HOXA5* promoter reduces its expression in the SAT pre-adipocytes of individuals with hypertrophic obesity, a well-established condition of restricted adipogenesis [13]. Besides its pathophysiological role, the methylation changes at the *HOXA5* locus may also act as biomarkers to predict the risk of obesity and type 2 diabetes (T2D), in line with the current view that methylation changes detected in bloodborne DNA may represent a mark for other tissues more directly implicated in disease pathogenesis [17]. Indeed, blood DNA methylation levels at the *HOXA5* gene were increased in subjects with a family history of T2D with hypertrophic obesity, and this was associated with increased adipose cell size, an independent predictor of T2D [13]. In obese individuals, blood DNA methylation at the *HOXA5* promoter strongly correlates with body mass index (BMI), indicating that the *HOXA5* methylation profile may also indicate the level of adiposity [13,18]. An intriguing feature of epigenetic changes is their dynamic nature and potential reversibility in response to targeted interventions [17], raising the possibility of developing a promising *HOXA5*-targeted therapeutic strategy.

Given the growing body of evidence linking *HOXA5* to the pathogenesis of obesity and T2D, a comprehensive review of its functions and mechanisms in regulating AT function is required. The potential application of *HOXA5* DNA methylation changes in the evaluation of disease risk and the efficacy of such an intervention will be discussed. A flowchart of the study selection process is presented in Figure S1.

## 2. *HOXA5*: A Crucial Transcription Factor in Development and Disease

The *HOXA5* gene encodes a 270-amino-acid protein with a highly conserved DNA-binding homeodomain, by which *HOXA5* finely regulates the mRNA expression of target genes determining cell and tissue identities during organism development [19].

The evidence for the essential role of *Hoxa5* in developmental and organogenesis comes mainly from the study of the *Hoxa5* knock-out mice [20,21]. While heterozygous mutants (*Hoxa5*^+/−^) were viable and indistinguishable from their wild-type littermates, the viability of homozygous mutants was markedly reduced with 50% of the mutant animals dying at birth or shortly thereafter from respiratory distress due to improper tracheal and lung morphogenesis [21]. *Hoxa5* absence leads to a significantly diminished expression of the *Nkx2-1* (*NK2 homeobox 1*) and *Foxa2* (*Forkhead box A2*), known key TFs governing lung epithelial cell differentiation and formation [22]. Survival *Hoxa5*^−/−^ mice develop an emphysema-like phenotype characterized by lung airspace enlargement [23]. Several *Hoxa5* mutant phenotypes closely resemble the phenotype in human lung pathologies. Consistently, altered *HOXA5* expression has been reported in patients with lung developmental diseases and adult pathologies [24,25,26]. These findings support the concept that genetic alterations affecting the *HOXA5* gene during early-life organogenesis may trigger an adult onset disease [17,27]. This is in line with recent evidence for the role of developmental genes in the origin of metabolic diseases, including obesity and altered body fat distribution [8,28]. *HOXA5* also regulates the function of the gastrointestinal tract. Indeed, its absence perturbs the gastric enzymatic activity and the cell specification of the gastric epithelium, resulting in a delay of intestinal acquisition of an adult-mode digestion [29]. Further, the lack of *Hoxa5* function alters thyroid development, as shown by smaller and disorganized follicles, causing transient hypothyroidism [30]. *HOXA5* has been shown to play a role in blood differentiation in humans. Indeed, sustained *HOXA5* expression in human hematopoietic progenitor cells causes a significant shift towards myeloid differentiation and away from erythroid differentiation [31,32]. Finally, *HOXA5* could substantially impact the immune microenvironment via the regulation of Th2 cells (T helper cells, also known as CD4^+^ cells, are a type of T cell that play an important role in the adaptive immune system by producing a variety of cytokines) and M2 macrophages (defined as alternative activated macrophages which are mainly involved in anti-inflammatory responses). Indeed, *Hoxa5* alleviates obesity-induced chronic inflammation by promoting M2 macrophage polarization in mouse AT [15,33,34]. In summary, perturbed cell proliferation, impaired differentiation, and altered remodeling of cell morphogenesis are common features in *Hoxa5* mutant mice [6].

Since the aforementioned pathophysiological processes are hallmarks of cancer cells, it is not surprising that deregulated *HOXA5* expression is associated with oncogenesis [35]. Nearly 70% of all breast carcinomas have decreased *HOXA5* expression levels [36], and its loss correlates with progression to higher-grade lesions. Indeed, in breast cancer, *HOXA5* exerts its tumor suppressor function by regulating p53 [37]. Reduced *HOXA5* expression is also a biomarker for poor prognostic in human non-small-cell lung cancer (NSCLC) [38]. Its overexpression significantly suppresses invasive capability in lung cancer by reducing the expression levels of several actin remodeling-related genes [39], a finding that is in agreement with the role of *HOXA5* in AT remodeling in obesity conditions [11]. Downregulation of the *HOXA5* gene in NSCLC can occur following *HOXA5* suppression by the *microRNA-196a* or due to aberrant promoter methylation [26,40]. Altered DNA methylation of *HOXA5* has also been linked to gastric tumorigenesis [41]. In colorectal cancer, the extent of *HOXA5* methylation is associated with its clinic-pathological characteristics [42]. According to the mainly suppressive nature of DNA methylation, the *HOXA5* gene is hyper-methylated and downregulated in colon cancer, and its re-expression induces loss of the cancer stem cell phenotype, preventing tumor progression and metastasis by inhibiting the β-catenin/WNT (*Wingless-related integration site*) signaling pathway [43]. The same situation has also been found in human pre-adipocytes, where *HOXA5* promoted adipogenesis by restraining *WNT* signaling [13]. Hyper-methylation of *HOXA5* and the subsequent reduction of its expression in leukemic cells correlate with a poor prognosis in chronic and acute myeloid leukemia [44,45]. Despite this well-established function as a tumor suppressor, several studies indicate that *HOXA5* may act as an oncogene in certain organs. Indeed, *HOXA5* expression is increased in gliomas, which is associated with increased tumor aggressiveness [46,47]. Thus, either a gain or a loss of *Hoxa5* gene expression can deregulate several signaling pathways, potentially triggering a transcriptional program in the hit tissues, disturbing normal development, and leading to tumorigenesis.

## 3. Role of *HOXA5* in Metabolically Unhealthy States

AT is now recognized as a highly active metabolic and endocrine organ impacting the whole-body energy homeostasis. However, it remains the primary site for energy storage and utilization. SAT is the largest adipose depot, storing around 80% of total body fat [48]. AT is composed of several cell types, including mature adipocytes, pre-adipocytes, and a range of inflammatory cells. The size of mature adipocytes has been widely used to link cellular and tissue functions to metabolic health. Healthy AT expands through a combination of hypertrophy (the enlargement of mature adipocytes) and hyperplasia (the recruitment and differentiation of pre-adipocytes). In obesity, AT may become severely dysfunctional and not expand properly to store the energy excess due to the inability to recruit new adipocytes through adipocyte differentiation and hypertrophic enlargement of pre-existing adipose cells. Moreover, after SAT has reached its limit for hypertrophic expansion, further caloric overload accumulates in ectopic locations, including key metabolic tissues like the liver and skeletal muscle. It has been largely demonstrated that these abnormalities result in hypertrophic obesity, local inflammation, and IR, increasing the risk of developing T2D [49,50,51].

An abnormal *HOXA5* expression in the AT, caused in part by epigenetic dysregulation, may impair normal development and differentiation programs resulting in malfunction at the cellular and tissue levels, which are primary abnormalities in obesity and its related illnesses. The role of *HOXA5* in the pathophysiology of metabolically unhealthy states such as AT hypertrophy, obesity, and T2D will be explored below.

### 3.1. HOXA5 and Hypertrophy of AT

Adipose cells develop from fibroblast-like pre-adipocytes to fully differentiated lipid-enriched adipocytes [51]. The transcriptional regulatory network controlling the maturation of adipocytes has been the focus of intense research and is reviewed elsewhere [52]. Here we discuss the central role of *HOXA5* in this process. *HOXA5* is highly expressed in AT [8], with expression induced early in pre-adipocytes, and continues to increase throughout the course of adipocyte differentiation in both humans and mice [11,13,53]. Gain or loss function of *Hoxa5* in murine pre-adipocyte cell models promoted or inhibited adipogenesis, respectively [11,54]. As in rodents, in human SAT pre-adipocytes, *HOXA5* depletion impaired adipocyte differentiation, which results in reduced lipid accumulation and decreased expression of key adipogenic markers [13].

While the requirement of *HOXA5* for adipogenesis is hereby conserved from mouse to human, the mechanisms responsible for its function in this process remain largely unknown. A first relevant contribution to this issue comes from our recent study where we reported that the silencing of *HOXA5* in human pre-adipocytes caused dysregulation of the transcriptional program at several genes in the *WNT*-signaling pathway, ultimately leading to impaired adipogenesis [13]. The pathophysiological relevance of these in vitro results was further supported by the findings that some of these *WNT* components, including the anti-adipogenic factors *NFATC1* (*Nuclear factor of activated T cells 1*) and *WNT2B* (*Wnt family member 2B*), were also significantly upregulated in SAT-derived pre-adipocytes of individuals with hypertrophic obesity, a well-established condition linked to restricted adipogenesis and characterized by low *HOXA5* expression [13,55,56]. In these subjects, remarkably, the *NFATC1* and *WNT2B* expression negatively correlated with that of *HOXA5*, supporting the negative regulation of *HOXA5* on the transcriptional activity of *NFATC1* and *WNT2B*. Considering that inappropriate activation of *WNT*-signaling prevents adipocyte differentiation and associates with hypertrophic adipocyte [57], it is possible that, in human pre-adipocytes, *HOXA5* promoted adipogenesis by restraining *WNT*-signaling, thus leading to healthy AT expansion. These observations are in keeping with in vivo rodent studies, where mice injected subcutaneously with adenovirus-*Hoxa5*-*shRNA* (*short hairpin RNA*) exhibited an AT characterized by hypertrophy of adipose cells, whereas *Hoxa5*-overexpressing mice featured reduced adipose cell size. Remarkably, the overexpression of *Hoxa5* resulted in a simultaneous reduction in body and AT weight, and improved insulin sensitivity. Additionally, the elevated expression of *Hoxa5* led to a decrease in triglycerides and levels of inflammatory cytokines. Conversely, *Hoxa5* silencing had adverse effects on the metabolic profile of the animals [34]. Further research has also indicated that *Hoxa5* promotes adipocyte differentiation by transcriptionally activating the key adipogenic factor *Fabp4* (*Fatty acid binding protein 4*) while inhibiting the *PKA/HSL (Protein kinase A/Hormone-sensitive lipase*) signaling pathway, whose dysregulation results in adipocyte hypertrophy [54,58]. Consistently, reduced *Fabp4* expression was detected in multiple *Hoxa5* null tissues [59]. In addition, both *HOXA5* and *FABP4* expression in human SAT were associated with elevated circulating triglycerides levels, which represent a feature of hypertrophic obesity [16,60]. Interestingly, Li Y et al. have demonstrated that *Hoxa5* induced the differentiation of pre-adipocytes by directly controlling the expression of *microRNA-574-5p*, whose downregulation in SAT is associated with adipocyte hypertrophy and IR in vivo [61]. Additionally, during hypertrophic obesity, the expansion of AT results in the activation of apoptotic signaling, including death receptor and mitochondrial pathways [62]. Notably, Feng F et al. have reported that a dysregulated expression of *Hoxa5*, upon palmitic acid exposure, induced apoptosis in mice adipocytes by altering the transcription activity of the pro-apoptotic gene *Bax* (*BCL2-associated X protein*), which is involved in the mitochondrial pathway [63]. These findings further demonstrate the regulatory role of *Hoxa5* in adipocyte function.

Despite the strength of these observations, several questions remain. We still have to comprehensively identify the direct targets of *HOXA5* that contribute to establishing the regulatory landscape during the promotion of adipocyte differentiation. Our unpublished data from transcriptome analysis of *HOXA5*-silenced human pre-adipocytes revealed that, during adipocyte differentiation, *HOXA5* could modulate a consistent set of genes, representing specific functional categories, including adipocyte metabolism, remodeling of adipocyte morphology, and a number of genes whose function in adipogenesis remains to be unearthed.

Thus, *HOXA5* stands out as an essential regulator of adipogenesis—both in vitro and in vivo—making it, along with its gene network, an intriguing molecular target for the treatment of AT dysfunction and associated metabolic diseases.

### 3.2. HOXA5 in Obesity and Type 2 Diabetes

As a crucial regulator of adipose cell development, *HOXA5* is required for proper lipid storage in the AT and may thus contribute to obesity. *HOXA5* is highly expressed in both visceral and SAT under physiologic conditions. Strikingly, *HOXA5* is strongly downregulated in both visceral and SAT from individuals who have obesity, and its expression is inversely associated with both BMI and waist-to-hip ratio (WHR) [8,28,64]. Animal studies, where we and others have demonstrated that the expression of *HOXA5* was significantly reduced in the abdominal fat depots of diet-induced and genetically obese mice, provide additional evidence for the role of *HOXA5* in the pathophysiology of obesity [8,11,65]. Although AT in visceral fat depot has long been considered a major contributor to the development of metabolic disorders, a growing body of evidence indicates that abdominal SAT depot also exerts a significant impact on the development of IR and T2D. Gene expression analysis of different SAT depots revealed that *HOXA5* expression was higher in abdominal SAT than in gluteal subcutaneous depots, and these same differences were observed in SAT fractions, including adipocytes and stromal-vascular fraction (SVF) cells. Thus, *HOXA5* expression is related to the degree of central obesity and the pattern of body fat distribution and, by extension, should be associated with an increased risk for obesity-related abnormalities such as IR and T2D. This is supported by the evidence from our recent study on lean subjects who were first-degree relatives of T2 diabetics (FDR), which are at high risk of developing the disorder. These subjects exhibit subcutaneous adipocyte hypertrophy due to restricted adipogenesis of resident pre-adipocytes, which make their abdominal SAT similar to those of obese subjects, even if not obese [66]. The storage capacity of their abdominal SAT is limited, and further caloric overload could promote ectopic fat accumulation, leading to local inflammation and IR fostering the onset of T2D. Remarkably, *HOXA5* expression was significantly downregulated in the pre-adipocytes of these subjects, and its reduced mRNA levels negatively correlated with the increased size of their subcutaneous adipocytes [9]. A downregulation of *HOXA5* was also observed in isolated adipocytes from the abdominal SAT of obese Pima Indians, a population with one of the highest prevalence rates of obesity and T2D [13,67]. Besides, individuals who have obesity and T2D showed reduced expression of *HOXA5* in abdominal SAT [61]. Therefore, the *HOXA5* deficit is a distinct feature of a dysfunctional abdominal SAT and may represent a marker of an increased risk of developing unfavorable metabolic outcomes, either in individuals with T2D familiarity or in subjects with obesity.

Changes in *HOXA5* expression have also been reported following weight/fat loss interventions. In a study investigating fat depot-specific transcriptome signatures before and after exercise-induced weight loss in obese African women, *HOXA5* was one of three genes with higher expression in abdominal vs. gluteal SAT in response to exercise training [68]. Furthermore, profound weight loss following bariatric surgery in obese individuals was accompanied by the upregulation of the *HOXA5* gene in the abdominal SAT [10]. Similarly, high-fat diet (HFD)-induced obese mice achieving significant weight loss after caloric restriction treatment revealed increased *HOXA5* expression in AT. These studies demonstrate that the expression of *HOXA5* is responsive to variations in fat mass upon weight loss interventions, further strengthening the functional relevance of this TF in AT by regulating multiple genes involved in AT remodeling [69]. It is also possible that the *HOXA5* transcription factor may control inflammation, such that its decreased AT expression during obesity may release the brake on pro-inflammatory genes, limiting tissue repair capability. A protective role of *Hoxa5* was reported in the studies of Cao W et al., which demonstrated that *Hoxa5* alleviates obesity-induced chronic inflammation by reducing pro-inflammatory cascades, including ER stress- and *TNC/TRL4/NF-κB* (*Tenascin-C/Toll-Like Receptor 4/Nuclear Factor Kappa B*) inflammatory pathways and, importantly, by promoting anti-inflammatory M2 macrophage polarization in mouse AT [34,70]. Similarly, forced *Hoxa5* expression attenuated carotid atherosclerosis-related inflammation in mice through enhancing macrophages switching toward the anti-inflammatory M2 phenotypes. In this context, *Hoxa5* exerted an anti-inflammatory function by regulating the *Med1* (*Mediator complex subunit 1*) gene [71], which also acts as a lipogenesis coactivator required for proper AT expansion [72]. These findings suggest that *HOXA5* promotes metabolically favorable AT remodelling in response to changes in body fat composition by suppressing inflammatory cascades while upregulating anti-inflammatory signals both in vitro and in vivo. The lungs of *Hoxa5* null mice consistently featured a perpetuated infiltration of activated macrophages [33,73]. Inflammatory pathways are being targeted as a novel treatment method to improve metabolic health [74]. Therefore, *Hoxa5* may be a potential molecular target for obesity and related metabolic disturbances. All the findings concerning the regulation of *HOXA5* in AT dysfunction from the aforementioned studies are summarized in Supplementary Table S1.

Obesity-related inflammation is widely acknowledged as a contributing factor in various cancers [75]. The presence of *HOXA5*, which possesses anti-inflammatory properties, plays a crucial role in regulating this inflammatory response. In obese individuals with insufficient *HOXA5* levels, this protective anti-inflammatory effect might be compromised. As a result, the chronic inflammatory environment that already exists in obesity could become even more pronounced, leading to a higher risk of cancer development and progression. Indeed, as in obesity, Pay P et al. reported that the loss of *HOXA5* increases the pro-inflammatory signaling pathway *NF-κB* to exacerbate breast cancer aggressiveness [76]. Therefore, *HOXA5* deficit may also affect multiple signaling pathways, commonly implicated in connecting obesity with an increased risk of cancer. Consistently, it was reported that the ectopic expression of *HOXA5* suppressed proliferation and neoplasia in cervical cancer cells by repressing *WNT* signaling [77]. In hypertrophic obesity, the silencing of *HOXA5* results in an improper activation of this pathway [13,78]. These findings indicate that deregulated *HOXA5* expression may serve as a molecular link between metabolic disorders and cancer development. Although this fascinating correlation holds great significance, it remains inadequately explored and warrants further attention.

## 4. Changes in *HOXA5* DNA Methylation Are Related to Metabolic Diseases

Epigenetic changes have been confirmed to play a critical role in the occurrence and progression of diverse metabolic diseases, including obesity and T2D. A deeper characterization of epigenetic regulatory mechanisms in metabolic diseases allows us to better understand these conditions and develop novel treatment strategies. Epigenetic regulation can occur at various levels, including DNA methylation, histone modifications, and noncoding RNA (ncRNA) modulation. They can all change the expression of genes without affecting their DNA sequence [75]. Histone modifications encompass intricate post-translational alterations like methylation (mono-, di-, and tri-methylation), acetylation, and SUMOylation. Post-translational changes in histone proteins can modify chromatin conformation and regulation of genes involved in glucose and lipid metabolism, thereby contributing to the phenotype of metabolic diseases [79]. NcRNAs have a significant impact on epigenetics, either by altering chromatin structure or controlling gene expression both at the transcriptional and post-transcriptional levels. A wide range of ncRNA types have been discovered, including microRNAs, long ncRNAs, circular RNAs, and novel small ncRNAs derived from known RNAs [80]. These ncRNAs are crucial for maintaining metabolic homeostasis. Dysregulated ncRNA expression is linked to the development and advancement of metabolic disorders [81]. The effects of histone modifications and ncRNA on the development of metabolic diseases have been extensively discussed elsewhere [82] and will not be covered in this review, which is focused on the role of DNA methylation in regulating the *HOXA5* gene in the context of metabolic disorders. DNA methylation is a crucial epigenetic modification involving the covalent addition of methyl groups to DNA bases. The process is governed by three conserved enzymes: DNA methyltransferase 1 (DNMT1), DNMT3A, and DNMT3B, which play a pivotal role in its occurrence and maintenance, contributing to normal development. Depending on its location, DNA methylation can lead to gene repression or activation. Typically, when it occurs near the transcription start site or enhancer regions, it suppresses gene expression [17]. The establishment and preservation of the DNA methylation pattern are influenced by various cellular environmental factors, including metabolism (Supplementary Figure S2). One critical pathway involved in this process is the methionine cycle, which converts methionine into S-adenosyl-methionine (SAM), a methyl donor for DNA. After donating the methyl group, SAM is converted back to homocysteine, which can follow two paths: remethylation and transsulfuration [83]. Proper regulation of these pathways is essential to support cellular health and fundamental biological processes. Investigating the transsulfuration pathway could serve as a promising approach to understanding changes in DNA methylation and predicting metabolic diseases, as clearly described by Werge MP et al. [83].

**Figure 1:**
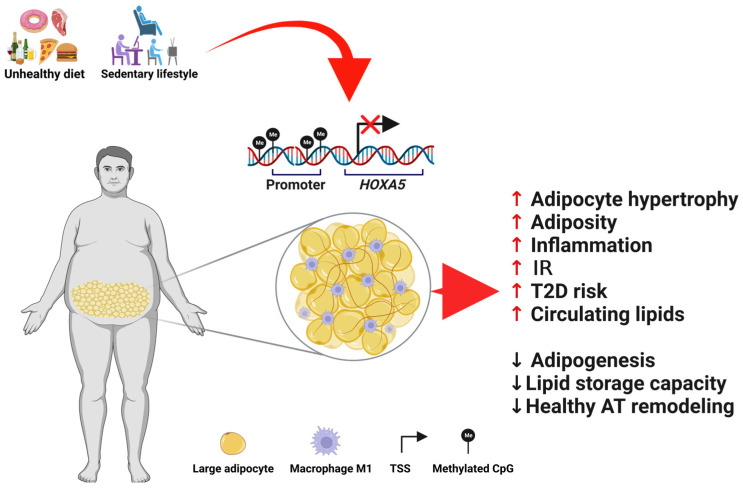
Altered *HOXA5* DNA methylation contributes to the pathogenesis of metabolic disturbances. Environmental factors, such as an unbalanced diet and a lack of exercise, can disrupt the correct make-up of DNA methylation at the *HOXA5* locus, whose deficit is a distinct feature of a dysfunctional abdominal SAT and may contribute to the development of adverse metabolic outcomes. Figure created with Biorender.com.

The transcription factor *HOXA5* is tightly epigenetically controlled throughout development [84], primarily by DNA methylation. Indeed, abnormal *HOXA5* promoter methylation is the most common way by which *HOXA5* is dysregulated in developmental diseases and several cancers [5]. In line with the current evidence that DNA methylation alterations in genes regulating AT function may be one of the crucial mechanisms linking obesity to its clinical complications, such as IR and T2D [50,85,86], the *HOXA5* gene has been shown to undergo dynamic changes in DNA methylation in metabolic diseases related to AT dysfunction. For example, in mice, a 5-month treatment with an HFD intake caused hyper-methylation of the *Hoxa5* promoter, which leads to its downregulation in abdominal fat. Interestingly, replacing HFD with a standard chow diet improves the metabolic phenotype while also restoring normal DNA methylation and expression levels at *Hoxa5*, suggesting that the *Hoxa5* methylation pattern may reflect the obesity response to nutritional intervention [11]. Likewise, *HOXA5* downregulation observed in pre-adipocytes of FDR individuals was caused by increased DNA methylation at its promoter region, which is significantly associated with signs of dysfunctional SAT, including adipocyte hypertrophy, that characterized these individuals [13]. This supports the concept that aberrant methylation at the promoter of this transcription factor could trigger a completely distinct transcriptional program in the hit tissue, namely, AT, resulting in a wide range of altered downstream transcriptional processes. A study by Rönn T et al., investigating the relationship between DNA methylation and triglyceride levels, found that in human SAT, the methylation of the *HOXA5* promoter was significantly associated with levels of circulating triglycerides, implying that these epigenetic changes may mediate the effects of triglycerides on IR [16] (Figure 1).

Besides its pathogenetic significance, the DNA methylation level of *HOXA5* may also serve as a potential biomarker to identify subjects at increased risk of metabolic disorders, in agreement with the current evidence that methylation changes detected in easily accessible tissues, like blood cells, may serve as a surrogate marker for major metabolic organs directly implicated in disease pathogenesis [87,88]. Indeed, as in pre-adipocytes, we found that the *HOXA5* promoter region is hyper-methylated in peripheral blood leukocytes of subjects with T2D familiarity. Importantly, this increased methylation was positively correlated with FDR adipose cell size in SAT, which has been demonstrated to predict T2D [13,89]. These findings indicate that *HOXA5* DNA methylation in the blood cells may represent a bona fide surrogate mark of a pre-adipocyte epigenetic profile in FDR. Our study further demonstrated the significance of the *HOXA5* methylation profile in blood as a mark of adipose tissue dysfunction and T2D risk since blood methylation at the *HOXA5* locus positively correlated with BMI in a group of obese individuals, indicating that the *HOXA5* epigenetic profile can also measure the degree of obesity [13,50]. Accordingly, a positive association between DNA methylation in *HOXA5* and BMI was reported in a Mexican American cohort, a population with a high prevalence of obesity. An EWAS study by Chilunga et al. examined the relationship between DNA methylation levels and IR in blood samples of an African American cohort, a population characterized by a high risk of developing IR and T2D [90]. Interestingly, in this study population, they found a close association between methylation levels at a specific CpG (*Cytosine-phosphate-Guanine*) site (cg14013695 at TSS of *HOXA5*) and IR, measured by the HOMA-IR index [91]. DNA methylation at the same *CpG* site has previously been associated with T2D-related traits in an EWAS of blood samples from Mexican Americans [92]. Together, these observations not only clearly demonstrate that DNA methylation at the *HOXA5* locus plays a role in the pathogenesis of obesity and its related disturbances, such as IR and T2D, but also suggest that the methylation status of *HOXA5* may be useful in clinical practice as an epigenetic biomarker in the prediction and diagnosis of metabolic diseases (Table 1).

## 5. *HOXA5* as a Potential Therapeutic Target

A further promising aspect of epigenetic changes lies in their dynamic nature and potential reversibility under targeted interventions [82]. This ambitious task might be accomplished by modifiable and lifestyle-sensitive epigenetic factors (e.g., nutritional factors, physical activity) or by using epigenetic modifying agents [17]. The FDA (Food and Drug Administration) has not yet approved any drugs targeting epigenetic pathways for the treatment of metabolic disorders. Nonetheless, several epigenetic drugs approved for other diseases, primarily cancer, may potentially work in metabolic diseases [82,93].

As DNA methylation is the primary mechanism by which *HOXA5* become dysregulated, research on drugs targeting methylation at the *HOXA5* locus appears promising for treating metabolic diseases. DNA methyltransferase inhibitors (DNMTi), which could modulate the methylation levels of specific genes, represent an attractive opportunity for metabolic disorders treatment. For example, the antiarrhythmic drug procainamide, which also acts as an inhibitor of DNMT1, protects against diabetes by restoring the expression of the homeobox *PDX1* (*Pancreatic and duodenal homeobox 1*) gene via a decrease in its methylation levels in β cells [94]. A similar strategy could be used to reduce the *HOXA5* promoter methylation in order to restore its function and restrain AT dysfunction in obesity. Another example comes from the decitabine, commonly known as 5-Aza-2′-deoxycytidine, which is a medication approved for the treatment of myelodysplastic syndromes [95]. Decitabine increases the expression of *PPARα* (*Peroxisome proliferator activated receptor alpha*) mRNA by regulating DNA methylation levels and reducing lipid accumulation, thereby alleviating non-alcoholic fatty liver disease [96].

Even more interestingly, this compound is also able to improve glucose homeostasis in T2D mice, in part by promoting macrophage polarization to the M2 anti-inflammatory phenotype via increasing *PPARγ* expression [97], suggesting that decitabine may also be applicable for diabetes therapy. Given the critical role of *HOXA5* in promoting the M2 macrophage switch in chronic inflammation, targeting its immunomodulatory action may provide therapeutic benefits and alleviate obesity-related inflammation and metabolic abnormalities. Furthermore, decitabine promotes osteogenic differentiation in a murine model of osteoporosis by decreasing the methylation levels of osteogenic genes, such as *RUNX2* (*Runt related transcription factor 2*). Notable, the expression of *RUNX2* is enhanced by *WNT2B*, which acts as an anti-adipogenic factor and is negatively regulated by *HOXA5* [13,98]. Although further research is needed, mainly regarding the adverse effects of these agents on off-target genes, epigenetic modifications specifically targeting *HOXA5* appear to be a promising strategy for precision medicine in metabolic diseases.

## 6. Conclusions

In this review, we highlighted the relevant role of the transcription factor *HOXA5* in the pathogenesis of metabolic diseases. *HOXA5* indeed has many faces. It is an essential regulator of adipocyte differentiation and promotes healthy AT expansion and distribution. *HOXA5* also ameliorates obesity-related inflammation and metabolic disturbance through its immunomodulatory function. Furthermore, changes in DNA methylation alterations at the *HOXA5* gene contribute to adipose cell dysfunction in obese and T2 diabetic individuals. Some of these epigenetic changes are also reflected in blood samples from people who are at high risk of or have metabolic disorders. This renders *HOXA5* a potential biomarker applicable to disease prediction and diagnosis. Given the susceptibility of epigenetic modifications to reversal through tailored interventions, targeting this transcription factor for therapeutic purposes might provide novel and realistic opportunities to prevent and treat metabolic diseases.

While this review presents compelling evidence showing that *HOXA5* deficiency is a molecular signature of AT dysfunction, a primary factor contributing to metabolic disorders, the exact causal relationship between *HOXA5* downregulation and the development of metabolic diseases is yet to be established. To achieve this, it is crucial to understand the specific target genes regulated by *HOXA5* that drive its metabolic effects. Additionally, creating an animal model with a targeted deletion of *HOXA5* specifically in AT holds promise as an exciting approach to further investigate its role in metabolic disease development.

## Figures and Tables

**Table 1:** Blood *HOXA5* DNA methylation as a biomarker for human metabolic diseases.

Position of DNA Methylation Change	Tissue	Disease and/or Clinical Manifestation	Reference
Promoter region	Peripheral blood leukocytes	Adipocyte hypertrophy; family history and risk of T2D	[13]
Promoter region	Peripheral blood leukocytes	Obesity; BMI	[13]
TSS 1500	Peripheral blood	Obesity; BMI	[18]
cg14013695	Whole blood	IR; HOMA-IR index	[91]
cg14013695	Peripheral blood cells	T2D risk	[92]

T2D, type 2 diabetes; BMI, body mass index; IR, insulin resistance; HOMA-IR, Homeostatic Model Assessment for Insulin Resistance; TSS 1500, the sequence region from −200 to −1500 nucleotides upstream of the Transcription Start Site; cg14013695, CpG site located within TSS 1500.

## Data Availability

Not applicable.

## Supplementary Materials

The following supporting information can be downloaded at: https://www.mdpi.com/article/10.3390/cells12162090/s1, Figure S1: Flowchart of the systematic review process; Figure S2: Metabolic pathways contributing to DNA methylation; Table S1: Regulation of HOXA5 in different cell types and adipose tissue depots under physiological and metabolically unhealthy conditions, in both humans and murine studies.
