# Supplementary material for: The Transcription Factor HOXA5: Novel Insights into Metabolic Diseases and Adipose Tissue Dysfunction
Source: Cells. 2023 Aug 18;12(16):2090. doi: 10.3390/cells12162090 (PMC10453582; doi:10.3390/cells12162090)
Supplement: Supplementary file 1 [file cells-12-02090-s001.zip › cells-2480785-supplementary.pdf]

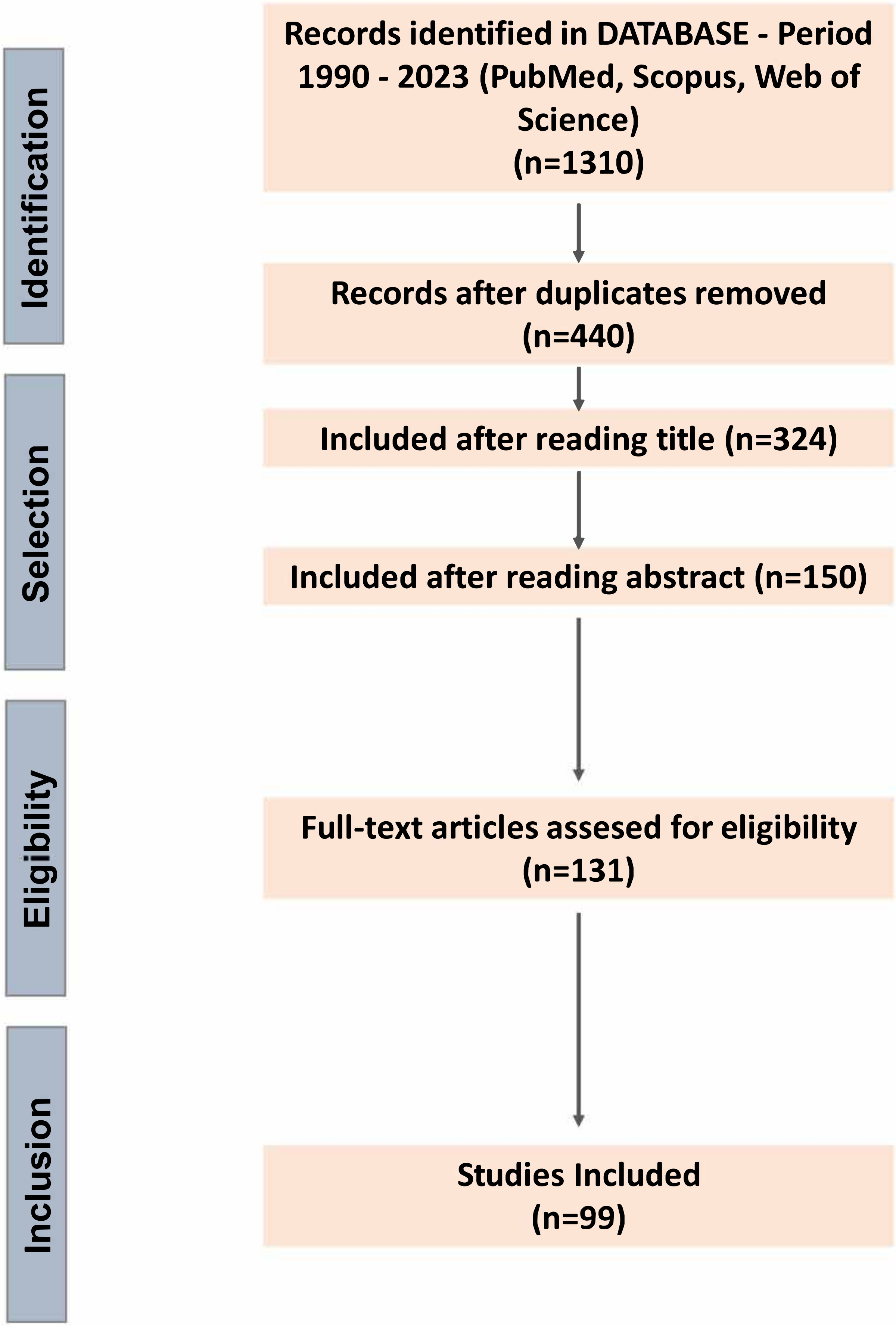

Figure S1. Flowchart of the systematic review process.

Supplementary Table S1. Regulation of *HOXA5* in different cell types and adipose tissue depots under physiological and metabolically unhealthy conditions, in both humans and murine studies.

Species	Physiologic/Metabolically Unhealthy State	Cell type/Tissue	HOXA5 gene expression	Reference
Human/Mouse	Adipocyte differentiation	3T3-L1 murine pre-adipocytes/adipocyte; pre-adipocytes/adipocytes from epi-WAT mouse and from human abdominal SAT	Increased in the course of adipogenesis	[11, 13, 53]
Mouse	High-fat diet induced Obesity	Whole Epi-WAT and Interscapular SAT	Decreased	[11, 65]
Mouse	Ob/Ob Mice	Epi- and perirenal WAT	Decreased	[65]
Mouse	High-fat diet induced Obesity	ATMs and adipocytes from epi- and inguinal WAT	Decreased	[34]
Mouse	$ApoE^{-/-}$ Mice	Carotid arteries from CAS mice; Macrophage line RAW264.7	Decreased	[71]
Human	Family history of T2D; Hypertrophic Obesity	Pre-adipocytes from Human abdominal SAT	Decreased	[13]
Human	Obesity	Whole Vis and SAT	Decreased	[8, 28]
Human	Lean healthy subjects	Whole Abdominal and gluteal SAT	Increased in abdominal *vs* gluteal SAT	[9]
Human	Obese with T2D	Whole Abdominal SAT	Decreased	[61]
Human	Obesity	Adipocytes from abdominal SAT	Decreased	[67]

Epi-WAT, epididymal white adipose tissue; SAT, subcutaneous adipose tissue; Ob/ob mice, leptin-deficient obesity mouse model; ATM, Adipose tissue macrophages; ApoE−/− mice, apolipoprotein E-deficient mouse model of atherosclerosis; CAS, Carotid atherosclerosis; Vis, visceral.

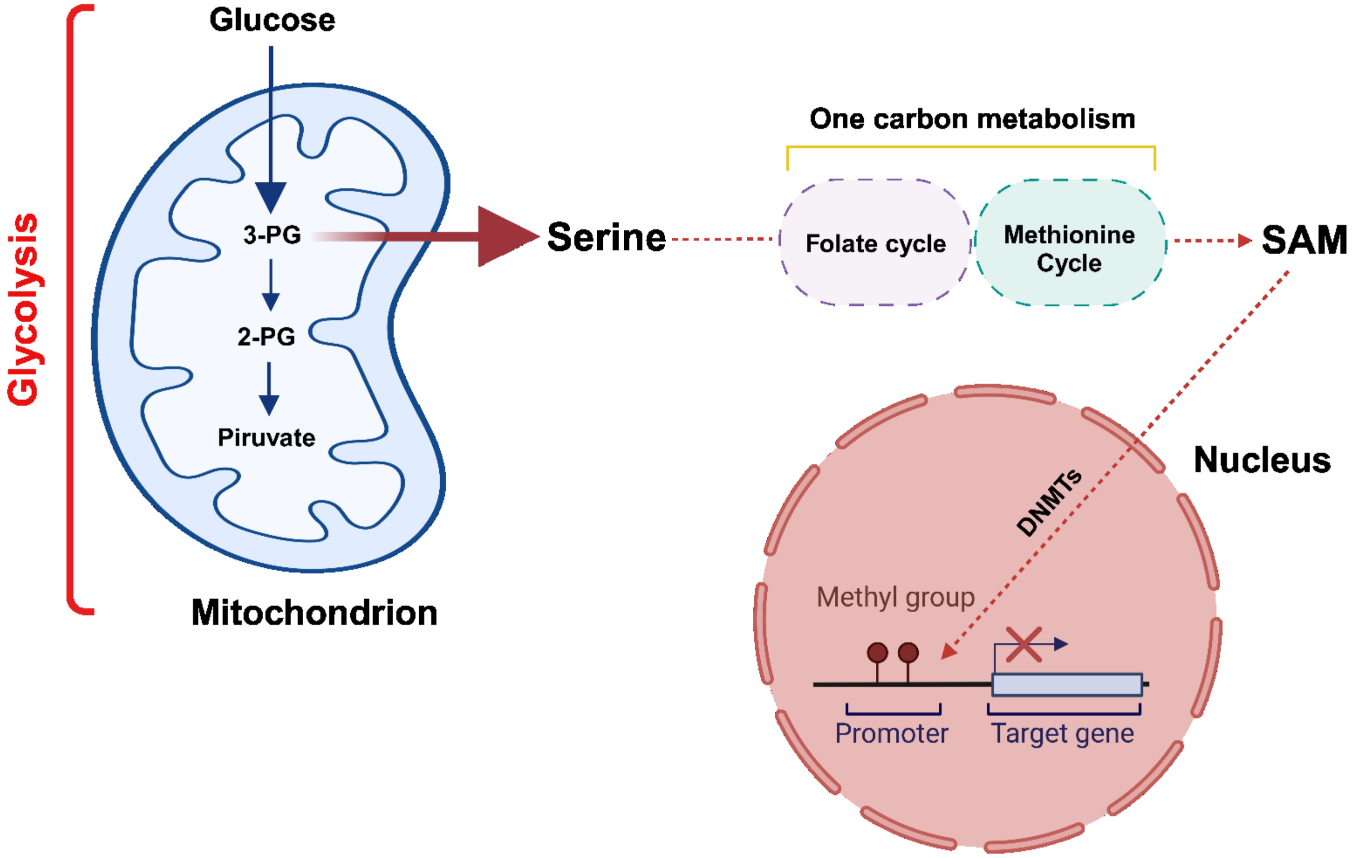

Figure S2. Metabolic pathways contributing to DNA methylation. Glucose-derived 3-phosphoglycerate (3-PG) is converted to serine, which is then metabolized through one-carbon metabolism (*i.e.*, folate and methionine cycles) to produce S-adenosylmethionine (SAM). SAM serves as the primary methyl donor for DNA methyltransferases (DNMTs) to add methyl groups to cytosine residues, forming 5-methylcytosine. Promoter DNA methylation is often associated with transcriptional repression. Figure created with Biorender.com
